# Meconium Stained Amniotic Fluid and Associated Factors among Women Who Gave Birth at Term in Adama Hospital Medical College, Ethiopia

**DOI:** 10.4314/ejhs.v33i2.6

**Published:** 2023-03

**Authors:** Tegene Dereje, Teshome Sharew, Lami Hunde

**Affiliations:** 1 Department of Obstetrics and Gynecology, Adama Hospital Medical College, Adama, Ethiopia; 2 Department of Public Health, Adama Hospital Medical College, Adama, Ethiopia

**Keywords:** Meconium stained amniotic fluid, Prevalence, Adama

## Abstract

**Background:**

Meconium stained amniotic fluid (MSAF) is a commonly observed phenomenon in day-to-day practice of obstetrics. The reported prevalence of MSAF was 7–22% of all term deliveries. Some of the factors that increases the risk of meconium stained amniotic fluid includes; advanced gestational age at delivery, prolonged rupture of membranes, intra-amniotic infection, pre-eclampsia, oligohydroamnios, and diabetes mellitus. The study aimed to determine the prevalence of meconium stained amniotic fluid and its associated factors among women who gave birth at term, from January 1^st^ to July 30^th^, 2020, at Adama Hospital Medical College.

**Methods:**

Institutional based cross-sectional study was conducted on 314 laboring women who gave birth at term. Systematic random sampling was used to select the study participants. Data entry and analysis were made by using Epi- info 7 and SPSS version 20, respectively.

**Results:**

The prevalence of meconium stained amniotic fluid was 23.9%. Late term pregnancy, Oligohydraminos, Antepartum hemorrhage, Premature rupture of membrane, and Non-reassuring fetal heart rate pattern were significantly associated with meconium-stained amniotic fluid.

**Conclusions:**

The prevalence of MSAF was comparable with other studies. Late-term pregnancy, oligohydramnios, antepartum hemorrhage, non-reassuring fetal heart rate pattern, and premature rupture of the membrane were factors associated with an increased risk of MSAF.

## Introduction

The fetus may pass meconium in to the amniotic fluid during pregnancy, leading to MSAF due to different reasons (1). The passage of meconium in utero has been described by various authors by different mechanisms. The three theories that have been suggested for fetal passage of meconium include; In response to fetal hypoxia, it may represent normal gastrointestinal tract maturation, and due to vagal stimulation (2, 3).

In utero, the passage of meconium is relatively common occurring in 7–22% of all term deliveries (4). The exact etiology of in utero passage of meconium remains unclear (5). Various risk factors that may cause stress on the fetus which lead to MSAF are: advanced gestational age at delivery, increased duration of rupture of membranes (ROM), the prolonged second stage of labor, intra-amniotic infection, placental insufficiency, maternal hypertension, pre-eclampsia, oligohydramnios, IUGR, GDM, overt diabetes mellitus, and maternal drug abuse (tobacco or cocaine) are associated with meconium staining of amniotic fluid (6–8).

Meconium-stained amniotic fluid (MSAF) is an alarming sign of fetal compromise and is associated with a poor perinatal outcome (9). Perinatal mortality increased in women with MSAF, even in those with a low risk of obstetric complications. Fetal and neonatal complications associated with MSAF include; increased risks of birth asphyxia and fetal distress, low birth weight, low Apgar scores, increased need for neonatal resuscitation, increased incidence of neonatal intensive care unit (NICU) admission, meconium aspiration syndrome, and early neonatal death. In deliveries complicated with MSAF, there is an increased risk of cesarean section, instrumental delivery, chorioamnionitis, and puerperal sepsis which results in higher morbidity and mortality in the mother (10–12).

Meconium-stained amniotic fluid is an alarming sign of fetal compromise and thus identifying the risk factors was very important for early identification and intervention. Since there was limited information about this issue in the study area, this study aimed to determine the magnitude of MSAF and its associated factors among women who gave birth at term in Adama hospital medical college (AHMC), Department of Obstetrics and Gynecology, from January 1^st^ to July 30^th^, 2020. The finding of this study can be used as valuable local evidence by clinicians and other healthcare providers to improve their practices.

## Methods and Materials

**The design and setting of the study**: A Hospital-based cross-sectional study was conducted at Adama Hospital Medical College (AHMC), Department of Obstetrics and Gynecology, Obstetrics ward from January 1^st^ to July 30^th^, 2020. AHMC is the only specialized Referral, and teaching hospital under the Oromia regional health bureau and is located in the Oromia region, Adama town which is located about 100 km southeast of the capital city, Addis Ababa, on the rail line to Djibouti. AHMC is one of the top ten governmental hospitals in Ethiopia. The Hospital provides different specialty services to over 6 million people coming from the catchment area. The average number of annual deliveries in the hospital was 9,000 deliveries. The Department of Obstetrics and Gynecology has a labor ward with 12 beds in the first stage room, 6 delivery couches in the second stage room, 2 operation tables, and 38 beds in the maternity ward. The department was staffed with 9 obstetrics and gynecology specialists, 51 midwives, and 4 clinical nurses.

**Inclusion and exclusion criteria**: All women who gave birth at term (gestational age between 37 and 41+6 completed weeks) in Adama hospital medical college (AHMC) during the study period were included in the study. Exclusion criteria include; unknown gestational age, preterm pregnancy (gestational age less than 37 completed weeks), post-term pregnancy (gestational age greater than or equal to 42 completed weeks), twins, triplets, and higher order gestation, mal-presentation (any fetal presentation other than vertex presentation), and fetal congenital anomalies were excluded from the study.

**Sample size and sampling procedure**: The sample size was calculated using a single population proportion formula by considering the prevalence of MSAF was 24.6 % from the previous study done in Felege Hiwot Referral Hospital, northwest Ethiopia (13). The required statistical assumptions for determining the sample size were a 95% level of confidence, a 5% margin of error, and a 10% non-response rate. Accordingly, 314 women were sampled for the current study. A systematic random sampling technique was used to select the required number of clients. The sampling frame was developed using the maternal medical record number registered on the delivery registration log book over 6 months (from January 1^st^ to July 30^th^, 2020). The total number of deliveries over 6 months was estimated as 4,500 which is divided by the sample size of 314 to determine the sampling interval which is K=14. For every 14 deliveries, one woman was selected. The first case was selected randomly by lottery methods. If the selected woman doesn't fulfill the inclusion criteria, the next woman will be chosen.

**Data collection tools and procedures**: Data were collected using a combination of interviews and chart reviews by three BSc midwives who were trained for this purpose. Structured interviewer-administered data collection formats were adopted and modified from different literature. Questionnaires that guide chart review and women interviews were structured into two logical sections (socio-demographic characteristics and obstetric-related factors). The questionnaire and consent form were first prepared in English and then translated into the Afan Oromo and Amharic languages, which are commonly spoken in the study setting. To check for consistency of the translation, the questionnaire and consent form was translated back into English. The questionnaire was pretested and checked for clarity and a logical sequence on 5% of the sample at Bishoftu Hospital before actual data collection began.

**Data management and analysis**: The collected data were coded and entered into Epi info version 7, then exported to SPSS version 20 for analysis. Data-processing tasks, such as data cleaning, categorizing, and transforming were then performed to make data ready for analysis. Descriptive analysis was performed to explore the characteristics of the study participants across their different socio-demographic and obstetric variables. Descriptive analyses like frequencies, the measure of central tendency, and cross-tabulations were performed. Logistic regression analysis was next used to identify the determinants of MSAF. The predictive model was developed using a standard model-building approach. In the process of building the model, first, a simple logistic regression analysis was performed to screen candidate variables that had a crude association with MSAF (P- value < 0.25). The selected candidate variables were then subjected to multiple logistic regression analysis to estimate their adjusted effects on MSAF. The statistical significance of independent variables in the final predictive model of MSAF was declared at a P-value < 0.05. Finally, the magnitude of association between the independent variables and MSAF was estimated using an odds ratio with a 95% confidence interval.

**Ethical consideration**: Ethical clearance & permission letter were obtained from the Institutional Review Board (IRB) of Adama Hospital Medical College. Informed written consent was taken from each participant after explaining the aim of the study. The name of the patient was not included in the data collection tool. Confidentiality was maintained during data analysis and interpretation.

## Results

**Socio-demographic characteristics**: A total of three hundred fourteen women were enrolled in the study with a response rate of 100%. The mean age of the study participants was 24.74 years with a standard deviation (SD) of ±4.23 years. One hundred thirty-two (42 %) of mothers were in the age group of 20 - 24 years and 188 (59.9%) of them were from urban areas. Of those respondents, 94.6% of the mothers were married and 53.8 % were housewives (Table 1).

**Table 1:** Socio-demographic characteristics of women who gave birth at term, from January 1^st^ to July 30^th^, 2020, in Adama Hospital Medical College

Characteristics		Number (%)
Age in years	≤ 19	36 (11.5)
	20 – 24	132 (42)
	25 – 29	118 (37.6)
	30 – 34	23 (7.3)
	≥ 35	5 (1.6)
Residency	Urban	188 (59.9)
	Rural	126 (40.1)
Religion	Orthodox	134 (42.7)
	Protestant	86 (27.4)
	Muslim	94 (29.9)
Educational Status	can't read and write	34 (10.8)
	Primary school	167 (53.2)
	Secondary school and above	113 (36)
Occupation	Housewife	169 (53.8)
	Merchant	94 (29.9)
	Employed	37 (11.8)
	Others	14 (4.5)
Marital Status	Single	10 (3.2)
	Married	297 (94.6)
	Divorced	7 (2.2)
Total		314 (100)

**Obstetrics-related characteristics**: The mean gestational age at delivery was 39.25 weeks with an SD of ±1.34 weeks. Nearly half of, 155 (49.4%) mothers were Para I and 308 (98.1%) had antenatal care follow-ups. The majority (87.9%) of mothers had spontaneous onset of labor and 86 (27.4%) had prolonged rupture of membrane. Thirty-seven (11.8%) of the mothers had an obstetric intervention for non-reassuring fetal heart rate patterns (NRFHP). Regarding the obstetric complications of respondents; 24 (7.6%), 15(4.8%), and 10(3.2%) had pregnancy-induced hypertension (PIH), Oligohydramnios, and antepartum hemorrhage (APH) respectively (Table 2).

**Table 2:** Obstetric characteristics of women who gave birth at term, from January 1^st^ to July 30^th^, 2020, in Adama Hospital Medical College

Obstetric Characteristics		Frequency (%)
Parity	Primipara	155 (49.4)
	Multipara	110 (35)
	Grand multipara	49 (15.6)
Gestational age in weeks	37 – 38 +6	262 (83.4)
	39 – 40 +6	25 (8.0)
	41 – 41+6	27 (8.6)
ANC follow up	Yes	308 (98.1)
	No	6 (1.9)
APH	Yes	10 (3.2)
	No	304 (96.8)
Oligohydraminos	Yes	15 (4.8)
	No	299 (95.2)
IUGR	Yes	15 (4.8)
	No	299 (95.2)
PIH	Yes	24 (7.6)
	No	290 (92.4)
PROM	Yes	19 (6.1)
	No	295 (93.9)
Onset of labor	Spontaneous	276 (87.9)
	Induced	38 (12.1)
Prolonged labor (≥ 12 hours)	Yes	229 (72.9)
	No	85 (27.1)
Prolonged ROM (≥ 8 hours)	Yes	86 (27.4)
	No	228 (72.6)
NRFHP	Yes	37 (11.8)
	No	277 (88.2)
Status of Liqour	Clear Liqour	239 (76.1)
	MSAF	75 (23.9)
Grade of Meconium	Grade – I	9 (12.0)
	Grade – II	36 (48.0)
	Grade – III	30 (40.0)
Stage of labor at the diagnosis of MSAF	Not in labor	3 (4.0)
	LFSOL	35 (46.7)
	AFSOL	26 (34.7)
	SOL	11 (14.7)
Mode of delivery	SVD	214 (68.2)
	C/D	87 (27.7)
	Instrumental delivery	13 (4.1)

**Prevalence of meconium-stained amniotic fluid**: The magnitude of meconium-stained amniotic fluid was 23.9% (95% CI: 19.1 – 29.3%). Out of 75 cases delivered with MSAF, 30 (40.0%) were grade 3 MSAF, 36 (48.0%) were grade 2 MSAF and 9 (12.0%) were grade 1 MSAF. Among women with MSAF 35 (46.7%) were in the latent first stage of labor (LFSOL) and 26 (34.7%) were in the active first stage of labor (AFSOL) at the time of diagnosis of MSAF.

**Factors associated with meconium-stained amniotic fluid**: Socio-demographic and obstetric factors associated with MSAF were identified using logistic regression analysis. First, a simple logistic regression analysis was used to identify candidate variables for a multiple logistic regression model. At this level: maternal age, Occupation Status, Gestational age, Onset of labor, Duration of rupture of membrane, Non-reassuring fetal heart rate pattern (NRFHP), Antepartum hemorrhage (APH), oligohydramnios, Pregnancy-induced hypertension (PIH), Intrauterine growth restriction (IUGR), premature rupture of membrane, and mode of delivery were selected as a candidate variable at P-value < 0.25.

All selected candidate variables were subjected to multiple logistic regression models to estimate their adjusted effect on MSAF after controlling for all possible confounding variables. Accordingly, there was a statistically significant association between MSAF and Gestational age at the time of delivery, Non-reassuring fetal heart rate pattern (NRFHP), Antepartum hemorrhage (APH), Premature rupture of membrane (PROM), and Oligohydramnos (p-value<0.05).

The study found that; late-term pregnancy (GA 41- 41+6 weeks) had 8.82 times higher odds of having MSAF than early-term pregnancy (GA 37 -38+6 weeks) (AOR = 8.82; 95% CI: 3.18 – 24.49). Compared to women with normal amniotic fluid volume, women with oligohydramnios had 5.09 times increased risk of developing MSAF (AOR = 5.09; 95% CI: 1.29 – 20.03). Mothers who had antepartum hemorrhage were 8.43 times more likely to develop MSAF during labor as compared to those who didn't have (AOR = 8.43; 95% CI: 2.02 - 35.17). Rupture of the membrane before the onset of labor (PROM) is associated with 10.06 times increased risk of MSAF, compared with a woman with the rupture of membrane after the onset of labor (AOR = 10.06; 95% CI: 1.27 - 79.98). Women who had Non-reassuring fetal heart rate patterns in labor had a 4.78 times higher risk of developing MSAF compared with women with normal fetal heart rate patterns (AOR = 4.78; 95% CI: 1.64 - 13.98) (Table 3).

**Table 3:** Factors association of meconium-stained amniotic fluid among women who gave birth at term, from January 1^st^ to July 30^th^, 2020, in Adama Hospital medical college

Variable		Status of Amniotic Fluid		COR (95% CI)	AOR (95% CI)
	
		Clear Liquor: Frequency (%)	MSAF: Frequency(%)		
Maternal age in	≤ 19	27(11.3%)	9 (12.0%)	Ref.	Ref.
years	20–24	108 (45.2%)	24 (32.0%)	0.67 [0.28,1.59]	0.49[0.19,1.30]
	25–29	87 (36.4%)	31 (41.3%)	1.07 [0.45,2.52]	0.67[0.25,1.80]
	30–34	16(6.7%)	7 (9.3%)	1.31 [0.41,4.21]	1.07[0.26,4.39]
	≥ 35	1(0.4%)	4 (5.3%)	12.0[1.18,121.81]*	7.24[0.56,93.44]
Occupation Status	House wife	131 (54.8%)	38 (50.7%)	Ref.	Ref.
	Merchant	68 (28.5%)	26 (34.7%)	1.32 [0.74,2.35]	0.99[0.49,1.99]
	Employed	32 (13.4%)	5 (6.7%)	0.54[1.96,1.48]*	0.48[0.15,1.55]
	Others	8 (3.3%)	6 (8.0%)	2.59[0.85, 7.91]*	1.69[0.42,6.87]
Gestational Age	37 – 38+6	214 (89.5%)	48 (64.0%)	Ref.	Ref.
	39 – 40+6	17 (7.1%)	8 (10.7%)	2.10[0.86, 5.14]*	1.29[0.41,4.05]
	41 . 41+6	8 (3.3%)	19 (25.3%)	10.60[4.38,25.61]*	8.82[3.18, 24.49]**
Antepartum	Yes	4 (1.7%)	6 (8.0%)	5.12[1.40, 18.62]*	8.43 [2.02, 35.17]*
Hemorrhage (APH)	No	235 (98.2%)	69 (92.0%)	Ref.	Ref.
Oligohydraminos	Yes	5 (2.1%)	10 (13.3%)	7.20[2.38, 21.81]*	5.09 [1.29, 20.03]*
	No	234 (97.9%)	65 (86.7%)	Ref.	Ref.
Pregnancy induced	Yes	12 (5.0%)	12 (16.0%)	3.60[1.54, 8.41]*	2.48[0.81,7.54]
hypertension (PIH)	No	227 (94.7%)	63 (84.0%)	Ref.	Ref.
IUGR	Yes	6 (2.5%)	9 (12.0%)	5.29[1.82,15.42]*	3.04[0.74,12.50]
	No	233 (97.4%)	66 (89.5%)	Ref.	Ref.
PROM	Yes	11 (4.6%)	8 (10.7%)	2.48[0.96, 6.40]*	10.06[1.27,79.98]**
	No	228(95.4%)	67(89.3%)	Ref.	Ref.
Onset of Labor	Spontaneous	213 (89.0%)	63 (84.9%)	Ref.	Ref.
	Induced	26 (10.9%)	12 (16.0%)	1.56[0.75, 3.27]*	0.20[0.03,1.20]
Prolonged ROM(.	Yes	61 (25.5%)	25 (33.3%)	0.68[0.39, 1.20]*	0.75[0.37,1.49]
8 hours)	No	178 (74.5%)	50 (66.7%)	Ref	Ref.
NRFHP	Yes	20 (8.4%)	17 (22.7%)	3.31[1.58, 6.52]*	4.78[1.64, 13.98]*
	No	219 (91.6%)	58 (77.3%)	Ref.	Ref.
Mode of Delivery	SVD	172 (72.0%)	42(56.0%)	Ref	Ref.
	C/D	60 (25.1%)	27 (36.0%)	1.84[1.05, 3.25]*	1.07[0.45,2.56]
	Instrumental	7 (2.9%)	6 (8.0%)	3.51[1.12,	1.32[0.27,6.53]
	Delivery			10.99]*	

Notes: *P < 0.25; **P < 0.05

## Discussion

The magnitude of meconium-stained amniotic fluid was 23.9% (95% CI: 19.1 - 29.3%). This finding was comparable with the findings of the study done in Felege-Hiwot Referral hospital, northwest Ethiopia (24.6%) (14). This might be due to the similarity in socio-demography of the health institutions, and quality of service provided. This finding was also in line with the finding from the Nigerian University Teaching Hospital (20.4%) (15). This might be due to the similarity in accessibility and quality of services. However, it was higher than the study finding in Pakistan (7.7%) (16) and Israel (10.9%) (17). This discrepancy might be due to the difference in the accessibility and the quality of services in study settings. In areas where there is better obstetric care, obstetric factors that increase the risk of MSAF in labor will be addressed properly so that the prevalence of MSAF will be low. On the other hand, this finding was lower than the study findings in IPGMER Hospital, India (30.6%) (18). The difference could be attributed to the time gap between the studies, which can reflect the difference in advancement in obstetric care and practice leading to variation in the prevalence.

In this study, gestational age at the time of delivery was significantly associated with MSAF in labor. Women in late-term pregnancy had 8.8 fold increased risk of MSAF, compared with women in early-term pregnancy. The findings of this study were similar to the study done in Idian which showed a significantly increased risk of MSAF in women whose gestational age is more than 40 weeks (12, 19). This might be explained by the maturation of the gastrointestinal tract and increased secretion of motilin by the fetus as gestational age advances which leads to increased fetal bowel peristalsis ending up in the passage of meconium.

The presence of oligohydramnios was another factor associated with the occurrence of MSAF. In this study, those women who had oligohydramnios were 5 times more likely to have meconium-stained amniotic fluid than those who didn't. Similar findings were reported in studies done in India and northwest Ethiopia (2, 20, 21). Fetuses with oligohydramnios usually had low or inadequate uteroplacental reserve as a cause of oligohydramnios, so when these women go into labor (started to have uterine contraction) there will be compression of the spiral arterioles with the uterine contraction, this leads to hypoxia in a fetus with underlying uteroplacental insufficiency. Fetal hypoxia results in the relaxation of the anal sphincter causing the passage of meconium into the amniotic fluid.

Women with PROM (premature rupture of membrane) had 10 fold increased risk of developing MSAF, compared with those with their counterparts. A similar finding was reported in the study done at SPHMMC(13) and the study conducted at Nigeria university teaching hospital (15). The increased risk of MSAF in women with PROM can be explained by; women with PROM having an increased risk of intrauterine fetal infection, which in turn causes fetal stress leading to the passage of meconium into the amniotic fluid.

Women with non-reassuring fetal heart rate patterns (NRFHRP) were nearly 4.8 times more likely to have meconium-stained liquor. This finding was consistent with studies done in Nigeria, India (Kolkata), and the United States of America (15, 17, 18, 22). It was also similar to the findings of the study done in Felege-Hiwot Referral Hospital, northwest Ethiopia. The explanation is often NRFHRP is a sign of hypoxia and hypoxia stimulates arginine vasopressin (AVP) release from the fetal pituitary gland and AVP stimulates the colonic smooth muscle to contract, resulting in intra amniotic defecation.

The limitation of this study was being a cross-sectional study, so it doesn't establish the temporal sequence between the exposure variable and the occurrence of MSAF. We recommend further studies with the other study design to have a better understanding of the temporal relationship between exposure variables and the occurrence of MSAF.

The prevalence of MSAF among mothers who gave birth at term was 23.9 %. In this study, we have seen that there was significantly increased risk of MSAF among women with late-term pregnancy, antepartum hemorrhage, oligohydramnios, premature rupture of membrane (PROM), and NRFHP. Due emphasis should be given by the health care providers during intrapartum follow-up of women with late-term pregnancy, oligohydramnios, APH, and PROM for earlier detection of MSAF in labor.
